# Anterior Cutaneous Nerve Entrapment Syndrome Presenting as Chronic Abdominal Pain

**DOI:** 10.7759/cureus.74921

**Published:** 2024-12-01

**Authors:** Holden W Pratka, Mike Martinez

**Affiliations:** 1 Anesthesiology, Baylor Scott and White Allsaints, Fort Worth, USA

**Keywords:** abdominal wall pain, anterior cutaneous nerve entrapment syndrome, chronic abdominal pain, interventional pain medicine, non-specific abdominal pain, spinal cord stimulation (scs)

## Abstract

Abdominal pain is one of the most common chief complaints that patients present with to healthcare facilities across specialties. Unfortunately for clinicians, the differential diagnosis for abdominal pain is vast. Abdominal pain can be broken down into two broad categories: visceral and non-visceral causes. One of the most common causes of non-visceral abdominal pain is anterior cutaneous nerve entrapment syndrome (ACNES). This report describes the case of a formerly active male in his 20s, who has been suffering from chronic abdominal pain for half of his life. Because of the broad differential and the overall lack of familiarity with ACNES, many patients face prolonged suffering and the psychological anguish of uncertainty. This report seeks to illuminate the importance of considering ACNES in a patient with chronic abdominal pain.

## Introduction

Patients presenting with chronic abdominal pain pose a wide differential for clinicians. Pain originating from the abdominal wall is often misdiagnosed as pain of visceral origin, which prolongs definitive treatment and patient suffering. Anterior cutaneous nerve entrapment syndrome (ACNES) is a common cause of chronic abdominal wall pain. ACNES has an estimated prevalence of 1 in 1,800 individuals [1]. At the population level, ACNES demonstrates a bimodal distribution, with patients commonly in the age ranges of 15 to 20 and 35 to 45 years [2]. However, as described in this report, it is important to note that there have been cases of pediatric presentations. Despite its prevalence, patients with this syndrome suffer from clinical confusion and diagnostic delays. The purpose of this presentation is to increase awareness of a common cause of abdominal pain to reduce diagnostic delays and shorten patient suffering.

## Case presentation

A well-developed male in his 20s presented with chronic abdominal pain localized to a discrete area in the right upper quadrant, adjacent to well-healed surgical scars. At the time of presentation, the patient had a previous diagnosis of ACNES and had undergone a neurectomy without success. The patient’s abdominal pain first began over 12 years prior with no specific inciting incident. Shortly after its onset, the pain spontaneously resolved, only to return a few years later. The patient was eventually diagnosed with ACNES and underwent a neurectomy five years after the initial presentation. He described this procedure as a brief success; however, the pain eventually returned in the same year. Prior to the onset of his pain, the patient was engaged in various sports and played guitar; however, this has since been limited due to his pain.

On examination, the patient demonstrated an antalgic gait but was otherwise neurologically intact. His mental status was depressed. Upon interviewing the patient, he discussed his depression, which he reported was being managed with fluoxetine by his primary care physician and, as needed, over-the-counter pain medication such as ibuprofen. It is well-documented that mood disorders and patient perception impact patient outcomes, particularly in the field of pain management [3]. Appropriate counselling and discussion with the patient was provided to discern the role this was playing in his clinical picture. The patient stated that the pain was persistent and exacerbated by physical activity. Remitting factors included heating pads and physical rest. He also sought alternative forms of treatment including physical therapy and chiropractors, with no success. Pertinent special testing included tensing the patient’s abdominal wall muscles, a maneuver known as the Carnett sign, as well as the pinch test, which involves reproducing the patient’s sense of discomfort by pinching the abdominal wall along a discrete area. These positive special tests further indicate a non-visceral source of his abdominal pain.

Being a diagnosis of exclusion, red flag symptoms such as a history of trauma, palpable masses, fevers, and changes in bowel habits were ruled out.

Following his diagnosis, a right unilateral transversus abdominis plane (TAP) block was performed under ultrasound guidance using 0.25% bupivacaine. The patient was also prescribed 1.8% topical lidocaine patches to be worn for 12 hours daily. He was seen in the clinic one week later to evaluate the success of the block and attested to near-complete relief. Between visits, the patient had worsening pain, necessitating a trip to the emergency department. In the emergency department, abdominal imaging was performed, which was inconclusive. He stated that his pain increased after the procedure but was unsure of the cause. His pain was still confined to the right upper quadrant; however, it had worsened. During this encounter, it was discussed in depth with the patient that it would be necessary to investigate non-physiologic causes for his pain as there could be a psychological element. He was agreeable to this and was referred for psychiatric evaluation. In the interim, he was prescribed cyclobenzaprine 10 mg tablets and was instructed to take one tablet three times a day for 30 days. A psychiatric evaluation was performed by another provider, and the results emphasized the need to proceed with investigating the physiologic causes of his pain. Given the patient's likely diagnosis of ACNES, we decided that utilizing a spinal cord stimulator to address neuropathic pain at the levels of T7-T12 was most appropriate. The role of spinal cord stimulators was discussed with the patient, and he agreed to proceed with this approach. At a subsequent visit, a spinal cord stimulator trial with two leads was performed. At his next visit a week later, he reported 80% improvement in pain since the spinal cord stimulator. On examination, the patient was ambulating well, and his mood had significantly improved. The temporary stimulator was removed, and the percutaneous spinal cord stimulator was implanted without complications. At a subsequent follow-up two weeks later, the patient attested to complete resolution in pain following spinal cord stimulator placement. His goals of becoming more active and playing guitar following spinal cord stimulator implantation were successful. He denied any pain except for occasional, sharp back pain that quickly dissipates. The surgical scars appeared to be well healed, and he denied any adverse outcome from the procedure. He appeared to have recovered well and was advised to return as needed in the future.

## Discussion

The pathophysiology of ACNES is rooted in the anatomy of the abdominal wall. The innervation of the anterior aspect of the abdominal wall arises from the T7 to T12 thoracic nerve roots. These nerve roots travel a tortuous path along their route to the lateral border of the rectus sheath. Because of this path, the T7 to T12 nerve roots are vulnerable to ischemic compression by the abdominal musculature, which can give rise to chronic abdominal wall pain [4].

The diagnostic work-up of abdominal pain begins with a thorough history and physical exam, which is oriented towards ruling out emergent and life-threatening causes of abdominal pain. Red flags that may arise during the examination of the patient include but are not limited to a plausible trauma history, fever, unstable vitals, rebound tenderness, guarding, bruising, and palpable masses. The diagnosis of ACNES involves soliciting these red flags and ruling out immediate threats to the patient’s well-being. Once these red flag symptoms have been ruled out, chronic causes of pain such as ACNES should be investigated. A hallmark ACNES is abdominal wall pain confined to a discrete area of the abdomen. This discrete area is most commonly around 2 cm in diameter and is typically unilateral and right-sided [5]. Additionally, the pain is typically worsened by physical activity that involves contraction of the abdominal musculature and is improved with rest.

Several authors have employed adjuncts to the physical examination such as ultrasound-guided TAP blocks to discern the origin of chronic abdominal wall pain. For example, Sahoo and Nair described two cases of suspected ACNES wherein ultrasound-guided TAP blocks aided both in the confirmation and amelioration of ACNES [6]. The use of TAP blocks as a diagnostic tool involves injecting a steroid alongside a neuromuscular blocking agent such as ropivacaine or bupivacaine between the internal oblique and transversus abdominis muscles. Following the procedure, if the patient attests to an improved pain level, this is an indirect indication that ACNES was in fact the diagnosis.

Additional studies have described the use of spinal cord stimulators in the treatment of ACNES. For example, a 2021 case series by Bral et al. described nine patients with ACNES who underwent placement of spinal cord stimulators. They reported that of these nine patients, all experienced improved pain ratings, with eight of the nine experiencing reductions greater than 50% as well as decreased pain medication use [7]. A similar yet slightly different approach to the management of ACNES includes the use of dorsal root ganglion stimulation (DRGS). Success with this modality has been demonstrated in a case series by Mol and Roumen [8]. This study demonstrated that out of five patients who underwent DRGS, three had greater than 50% pain relief at the 12-month follow-up. This particular patient is scheduled to be seen at the 12-month mark and then on an as-needed basis to assess the spinal cord stimulator. While DRGS offers an advantage over spinal cord stimulators in that they can be utilized to target a more selective area, there are well-documented risks that may make this option less favorable, such as the higher risk of nerve injury that is secondary to lead placement near the dorsal root ganglion [9].

## Conclusions

Abdominal pain originating from the abdominal wall is prevalent but is often misdiagnosed as pain of visceral origin. Diagnostic delays compound and prolong patient suffering but can be minimized by increasing awareness of ACNES. This patient continues to report greater than 80% improvement in discomfort following placement of a spinal cord stimulator. While this intervention was crucial to his pain management regimen, the role of appropriate patient counseling cannot be understated. It is crucial to understand and address the role of mood disorders and patient perception in pain management.
